# 

**Published:** 1840-09

**Authors:** 


### CONTENTS

OF NO. IX.

### ORIGINAL COMMUNICATIONS.

### ESSAYS AND CASES.

ART. I.—Thoughts on the action and influence of the Nervous System, and on the means of strengthening and improving them. By CHARLES CALDWELL, M.D. - - 165

### REVIEWS.

ART. II.—Elements of Pathological Anatomy, illustrated by numerous engravings. "In Morbis, sive acutis, sive chronicis, viget occultum, per humanas speculationes fere incomprehensible."--Baglivi. By SAMUEL D. GROSS, M.D. Late professor of General Anatomy, Physiology, and Pathological Anatomy, in the Medical Department of the Cincinnati College. Vols. II, 8vo., Boston, 1839. Marsh, Capen, Lyon & Webb and James B. Dow. - 187

ART. III—Guy's Hospital Reports. No. viii. April, 1839. Edited by GEORGE H. BARLOW, M. A., &c., and JAMES P. BARRIGTON, M. A. 8vo. pp. 263. London, 1839. 195

Analyses are given of the following papers:

1st. On the Disorders of the Brain, connected with Diseased Kidneys. By THOMAS ADDISON, M.D. - - - - - - - - 196

2d. On Perforations of the Stomach from Disease and Poisoning. By ALFRED S. TAYLOR. - - - - - - - - 201

3d. On the diurnal variations of the Pulse. By WILLIAM AUGUSTUS GUY, M. B. H. - - - - - - - - - 210

4th. Observations on Poisoning, by the Vapours of Burning Charcoal and Coals. By GOLDING BIRD, M.D., H. - - - - - 215

5th. Two cases of Poisoning by the Inhalation of Carburetted Hydrogen. By THOMAS P. TEALE, F. L. S. - - - - - 220

6th. Case of Imperforate Uterus. By ALEXANDER TWEEDIE. - - 224

7th. On Incision in Cases of Occlusion and Rigidity of the Uterus. By SAMUEL ASHWELL, M.D. - - - - - - - - 227

SELECTIONS FROM AMERICAN AND FOREIGN JOURNALS.

On the employment of a new remedy, the *Monesia*, in Menorrhagia, Diarrhœa, &c. - - - - - - - - - - - 231

ORIGINAL INTELLIGENCE.

The Natchez Tornado. - - - - - - - - - 237

Nasal Polypus cured with Sanguinaria Canadensis. - - - 237

Stramonium in incipient Trismus. - - - - - - - 239

Family Cataract. - - - - - - - - - - - 238

The effects of Camphor on Vegetables. - - - - - - 239

